# Risk and protective factors for anxiety during COVID-19 pandemic

**DOI:** 10.1186/s12889-021-11118-8

**Published:** 2021-06-04

**Authors:** Jingyi Zhong, Chenghui Zhong, Lan Qiu, Jiayi Li, Jiayi Lai, Wenfeng Lu, Shuguang Wang, Jiacai Zhong, Jing Zhao, Yun Zhou

**Affiliations:** 1 State Key Laboratory of Respiratory Disease, The First Affiliated Hospital of Guangzhou Medical University, Guangzhou, 510120 China; 2 School of Public Health, Guangzhou Medical University, Guangzhou, 510120 China

**Keywords:** Risk factors, Protective factors, Anxiety, Score, COVID-19

## Abstract

**Background:**

Coronavirus Disease 2019 (COVID-19) is a global pandemic and an anxiety-provoking event. There are few studies to identify potential risk and protective factors related to anxiety during COVID-19 pandemic.

**Methods:**

We collected information on demographic data and lifestyles by a web-based survey of 19,802 participants from 34 provinces in China during COVID-19 pandemic. Level of anxiety was evaluated using the Self-Rating Anxiety Scale. We used ordinal multivariable logistic regression to estimate the associations of anxiety level with potential risk and protective factors. We further developed a new score to simplify the assessment of anxiety during COVID-19 crisis.

**Results:**

Among 19,802 participants, we found that those who were front-line medical personnel, suffered from chronic disease, with present symptoms of SARS-CoV-2 infection or contact history had 112, 93, 40 and 15% increased risk of higher anxiety level; while those with knowledge about personal protective measures or wore masks had 75 and 29% lower risk of higher anxiety level respectively. We developed a risk score by calculating the sum of single score of 17 factors. Each one increase of the risk score was associated with a 297% increase in anxiety index score. In categorical analysis, low risk (the risk score between 1 to 2), the moderate risk group (the risk score of 3) and high risk group (the risk score ≥ 4) had − 0.40 (95% CI: − 1.55, 0.76), 1.44 (95% CI: 0.27, 2.61) and 9.18 (95% CI: 8.04, 10.33) increase in anxiety index score, and 26% (95% CI: − 7, 72%), 172% (95% CI: 100, 270%), and 733% (95% CI: 516, 1026%) higher risk of anxiety respectively, when compared with the very low risk group (the risk score of 0). The AUC was 0.73 (95% CI, 0.72, 0.74) for the model fitted the developed risk score, with the cut-off point of 3.5.

**Conclusions:**

These findings revealed protective and risk factors associated with anxiety, and developed a simple method of identifying people who are at an increased risk of anxiety during COVID-19 pandemic.

**Supplementary Information:**

The online version contains supplementary material available at 10.1186/s12889-021-11118-8.

## Background

COVID-19 outbreak that occurred since December 2019 has become one of the greatest threats to global public health. According to the report from the World Health Organization (WHO), more than 127 million people across the globe have been infected, causing 2.7 million deaths since January, 2020 [1]. The COVID-19 pandemic and resulting economic downturn have negatively affected mental health. In a recent Kaiser Family Foundation (KFF) poll, 45% of US adults reported mental health problems due to worry and stress during the COVID-19 crisis [2]. Previous studies suggested that the prevalence of depressive symptoms was 20.1% in a Chinese population during the first month of a widely implemented quarantine due to COVID-19, which is much higher than previous reports on the lifetime rate of depressive symptoms (6.8%) [3]. Previous studies have reported that the Chinese have a wide range of mental health problems during the COVID-19 pandemic, such as depression, stress, panic, anger, insomnia, PTSD, and suicidal behavior [4–7].

Anxiety is a prominent mental health problem that occurred in disaster events [8]. Compared to a natural disaster or welfare event (i.e., earthquake or terrorist attack), disaster events from emerging infectious diseases might cause anxiety not only due to the extremely high morbidity and mortality, but also due to the measures taken to secure public health. For example, isolation, quarantine, social distancing and community containment may lead to negative social and economic consequences on communities as well as public health worries [9]. Persistently mental disorders may cause post traumatic stress disorder (PTSD) or acute stress reaction (also known as acute stress disorder) after life-threatening events, or adjustment disorder triggered by an identifiable and stressful life change [10]. Therefore, protective interventions on anxiety are necessary during the COVID-19 crisis when we focus on the treatment and control of physical damage caused by SARS-CoV-2. Assessing the risk and protective factors that contribute to anxiety helps practitioners select appropriate interventions. Previous studies have reported that front-line medical personnel, chronic disease, and contact history were associated with increased risk of anxiety during an epidemic [11–13], but limited evidence for COVID-19 to date.

Therefore, we conducted a web-based study to collect information on demographic data and lifestyles, and to assess the levels of anxiety among 19,802 participants in China during the early outbreak of COVID-19. Associations between potential factors and mental health were estimated to identify the risk and protective factors. We further calculated a score of multivariate factors to assess the effect of combinations of multivariate factors on anxiety.

## Methods

### Study population

We used Sojump, a professional online questionnaire survey platform to collect information on demographics, lifestyles and risk factors for COVID-19.This study was conducted among 20,102 participants from 34 provinces in China during January, 2020 by a web-based investigation. All participants included in this analysis were all recruited according to the following inclusion criteria: 1) residents aged 14–55 years, who can fully understand the questions; 2) those who could use a smartphone to complete the standardized questionnaires voluntarily participated in this study; 3) those who answered the questionnaire for more than 100 s. After excluding those who reported invalid information on date, such as the date before the outbreak of COVID-19 or beyond the date of filling out the form, whose data was unable to ensure its authenticity, or those with outliers on age (< 1% or > 99%), therefore 19,802 participants included in the final analysis. To fill in the form, the subjects were first asked to clearly state that they agreed to participant in the investigation. All participants provided written informed consent.

### Variates

Variates were collected by a web-based investigation, covering information on demographic and socioeconomics, lifestyles including age, sex, body mass index (BMI), race, smoking status, drinking status, chronic diseases (including hypertension, hyperlipidemia, diabetes, asthma, chronic obstructive pulmonary disease (COPD), chronic bronchitis, heart disease, gout, thyroid nodules, thyroid cancer, and lung cancer), and present symptoms of SARS-CoV-2 infection (including fever, cough, runny nose, sore throat, shortness of breath, fatigue, nasal congestion, headache, vomiting and diarrhea), and regular physical activity, etc. Regular physical activity was defined as exercise regularly within the recent six months [14]. Current smoker was defined as an adult who has smoked 100 cigarettes in his or her lifetime and who currently smokes cigarettes, and former smoker was defined as an adult who has smoked at least 100 cigarettes in his or her lifetime but who had quit smoking at the time of interview. Individual who had never smoked, or who had smoked less than 100 cigarettes in his or her lifetime was defined as non-smoker [15]. Current drinker was defined as at least 12 drinks in past year; former drinking was defined as any one year in lifetime but no drinks in past year; while non-drinker was defined as fewer than 12 drinks in lifetime [16]. BMI was calculated by dividing self-reported weight in kilograms by height in meters squared. Each participant’s chronic disease history information was collected by asking the question “Have you ever been diagnosed with any diseases including hypertension, hyperlipidemia, diabetes, asthma, chronic obstructive pulmonary disease (COPD), chronic bronchitis, heart disease, gout, thyroid nodules, thyroid cancer and lung cancer?”

### Anxiety status assessment

We used Self-Rating Anxiety Scale (SAS), a self-report scale developed by Zung [17], to assess anxiety symptom using 20 self-report items. There are 15 items worded symptomatically positive rated on a 4–1 scale (“a little of the time,” “some of the time,” “good part of the time,” and “most of the time”), and 5 items symptomatically negative rated on a 1–4 scale. A standardized scoring algorithm is used to define symptoms of anxiety, with an original raw score range of 20–80. The original raw score cut-off of 40 we used would be most appropriate when the SAS is used in research [18, 19]. The raw score is then converted to an index score by multiplying 1.25. In the Chinese public, the index score has the following 4 categories [4]: the index score of “< 50,” “50–59,” “60–69,” and “≥ 70” were defined as “normal,” “mild anxiety,” “moderate anxiety,” and “severe anxiety.” The scale also showed high internal consistency and good reliability (Cronbach’s alpha was .95).

### Statistical analysis

We applied summary statistics to describe baseline characteristics and anxiety level of all participants. Ordinal multivariable logistic regression analyses were performed to estimate various risk and protective factors. We entered all variables in the first logistic regression model. Odds ratio (OR) and 95% confidence interval (CI) of anxiety associated with significant factors in the first model were estimated in the second logistic regression model. Points were attributed to the variables in the second model. The risk score was calculated by summing up the single score of each factor, which can also present the number of anxiety related factors for each participant (i.e. one who had the risk score of three means that they had three anxiety-related factors). The risk scores were grouped into scores of 0 (very low risk), 1–2 (low risk), 3 (moderate risk), and ≥ 4 (high risk).

An analysis of variance (ANOVA) and a chi-square test were used to compare the average anxiety index score and percentage of anxiety of the four groups. Both linear regression models and logistic regression models were used to estimate the association of developed risk score with anxiety index score and the risk of anxiety respectively. To further investigate whether the developed risk score can predict anxiety, we calculated the area under curve (AUC). *P*-values were 2-sided and considered statistically significant at less than .05. Analyses were performed by SPSS version 22.0 (https://www.ibm.com/products/software, RRID: SCR_002865), and image analyses were conducted with R Project for Statistical Computing version 3.6.0 (http://www.r-project.org/, RRID:SCR_001905), RStudio version 4.0.2 (http://www.rstudio.com/, RRID:SCR_000432), Microsoft Excel version 2019 (https://www.microsoft.com/en-gb/, RRID:SCR_016137) and ArcMap version 10.2 (https://desktop.arcgis.com/zh-cn/arcmap/).

### Patient and public involvement

Because this study used existing epidemiological data, it was not appropriate to involve patients or the public in the research.

## Results

### Participant characteristics

**Fig. 1:**
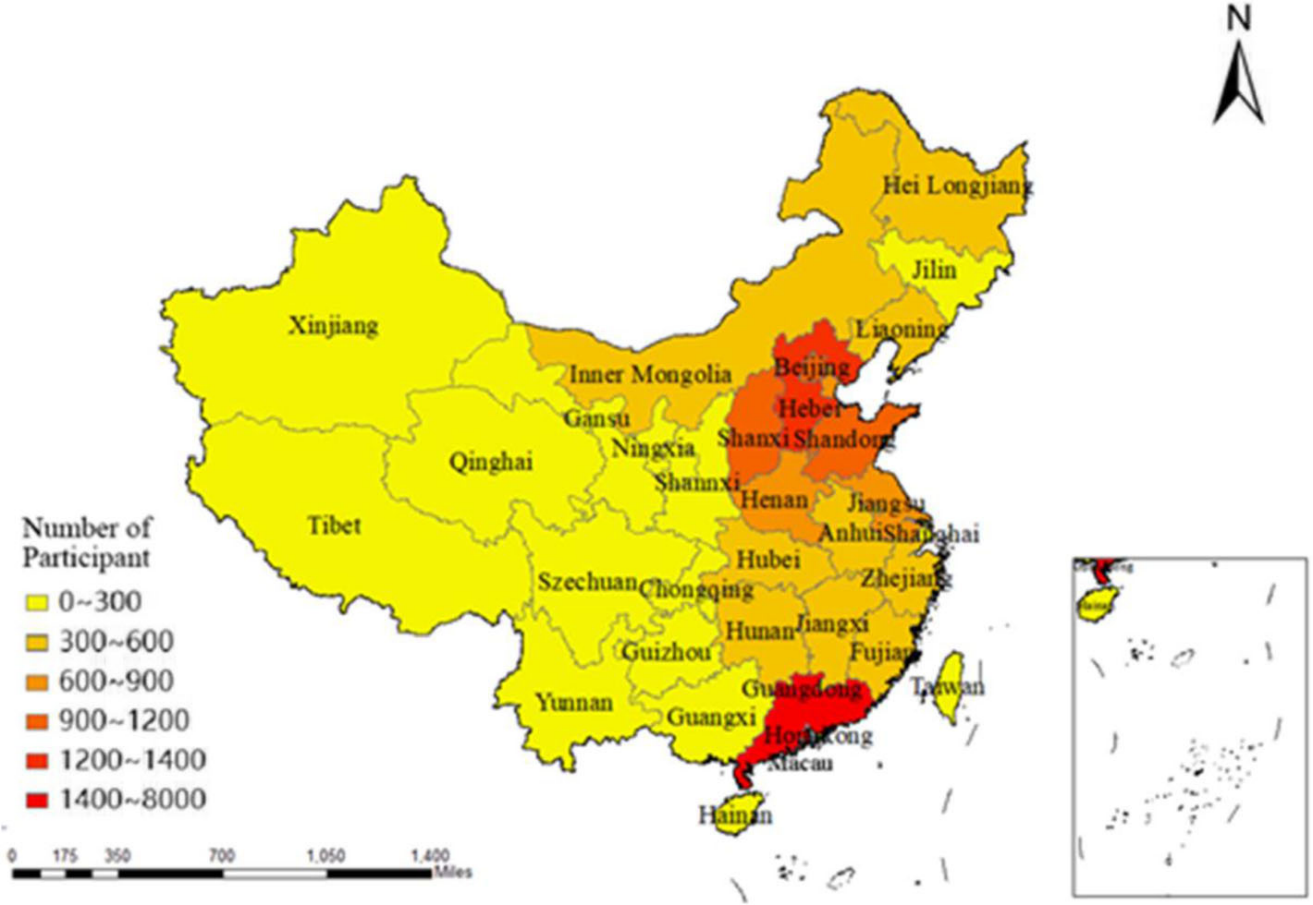
Distribution of Participants. Different colors represent the participants in different provinces

All participants were from 34 provinces and approximately a half (*n* = 10,459) were from Guangdong Province, Hebei Province, and Shanxi Province (Fig. 1). The mean (SD) age of the 19,802 participants were 25.3 (8.1) years, ranging from 14 to 55, including 51.1% (*n* = 10,121) men, 4.9% (*n* = 964) front-line medical personnel, 10.6% (*n* = 2096) self-employed, 2.8% (*n* = 558) with chronic disease, 15.2% (*n* = 3004) with contact history, 87.5% (*n* = 17,334) are characterized as non-smokers, 73.9% (*n* = 14,629) as non-drinker, and 2.2% (*n* = 436) exposed to wild animals. Characteristics and anxiety levels of the participants were summarized in Tables 1 and 2. Overall, 15,277 (77.1%) participants were without anxiety, while 2157 (10.9%), 1268 (6.4%) and 1100 (5.6%) participants had mild, moderate, and severe levels of anxiety respectively. Characteristics of participants with different levels of anxiety are also presented in Table S1.

**Table 1:** Characteristics of participants (*n* = 19,802)

Characteristics, *n* (%)	All participants (*n* = 19,802)
Age, year	
14–24	11,630 (58.7)
25–35	5746 (29.0)
36–55	2426 (12.3)
Male	10,121 (51.1)
Body mass index, kg/m^2^	
< 18.5	4232 (21.4)
18.5–23.9	11,553 (58.3)
> 23.9	4017 (20.3)
Race	
The Hans	19,075 (96.3)
Other	727 (3.7)
Smoking status^a^	
Current smoker	1704 (8.6)
Former smoker	764 (3.9)
Non-smoker	17,334 (87.5)
Drinking status^b^	
Current drinker	3057 (15.4)
Former drinker	2116 (10.7)
Non-drinker	14,629 (73.9)
Job	
Student or employee	16,648 (84.1)
Self-employed	2096 (10.6)
Retired and unemployed	1058 (5.3)
Front-line medical personnel	964 (4.9)
In Hubei Province in the past month	1016 (5.1)
Meeting relatives or friends coming from Hubei in the past month	1159 (5.9)
Quarantine^c^	1175 (5.9)
Exposure to wild animals	436 (2.2)
Gatherings & meetings^d^	6027 (30.4)
Wearing masks	17,621 (89.0)
Regular physical activity^e^	10,952 (55.3)
Suspicion of SARS-CoV-2 infection	571 (2.9)
Contact history^f^	3004 (15.2)
Knowledge about personal protective measures	15,418 (77.9)
Present symptoms of SARS-CoV-2 infection^g^	827 (4.2)
Chronic disease^h^	558 (2.8)
Chronic disease classification, n (%)	
Respiratory disease and cardiovascular disease	49 (0.2)
Simple respiratory disease	111 (0.6)
Simple cardiovascular disease	251 (1.3)
Other	147 (0.7)
Without chronic disease	19,244 (97.2)

^a^Current smoker was defined as an adult who has smoked 100 cigarettes in his or her lifetime and who currently smokes cigarettes; former smoker was defined as an adult who has smoked at least 100 cigarettes in his or her lifetime but who had quit smoking at the time of interview; while non-smoker was defined as an adult who has never smoked, or who has smoked less than 100 cigarettes in his or her lifetime

^b^Current drinker was defined as at least 12 drinks in the past year; former drinker was defined as at least 12 drinks in any one year in lifetime but no drinks in past year; while non-drinker was defined as fewer than 12 drinks in lifetime

^c^Been or are in quarantine for this outbreak, including mandatory isolation and self-isolation at home/hotel

^d^Been to a company meeting or a family dinner in the last two weeks

^e^Regular physical activity was defined as regular exercise within the recent six months

^f^Close contact with a confirmed or suspected case of COVID-19 without taking precautions

^g^Including fever, cough, runny nose, sore throat, shortness of breath, fatigue, nasal congestion, headache, vomiting and diarrhea

^h^Including hypertension, hyperlipidemia, diabetes, asthma, chronic obstructive pulmonary disease (COPD), chronic bronchitis, heart disease, gout, thyroid nodules, thyroid cancer, and lung cancer

**Table 2:** Anxiety Level of All Participants (*n* = 19,802)

Self-Rating anxiety scale	All participants (*n* = 19,802)
Anxiety index score, mean (SD)	39.60 (14.78)
Classification (anxiety index score), *n* (%)	
Normal (< 50)	15,277 (77.1)
Mild (50–59)	2157 (10.9)
Moderate (60–69)	1268 (6.4)
Severe (≥ 70)	1100 (5.6)

### Risk and protective factors associated with anxiety level

There are 17 factors with significance in the first multivariate logistic regression model (Table S2). In the second multivariate logistic regression analysis, we found that participants aged 25–35 years, males, former smokers or drinkers, or those who were front-line medical personnel, self-employed, exposed to wild animals, or had chronic disease, suspicion of SARS-CoV-2 infection, present symptoms of SARS-CoV-2 infection, regular physical activity, contact history, or met relatives or friends coming from Hubei in the past month were significantly associated with 48, 40, 21, 17, 112, 62, 31, 93, 66, 40, 37, 15, 23% respectively increased risk of anxiety, while those aged 14–24, wore masks or had knowledge about personal protective measures were associated with 33, 29, 75% respectively decline in risk of anxiety (Table 3).

**Table 3:** Analyses of the Association Between Characteristic and Anxiety Level (*n* = 19,802)

Variable	*t*-value	*df*	Adjusted odds ratio (95% CI)^a^
Age, year			
14–24	46.64	1	0.67 (0.59, 0.75)^**^
25–35	47.49	1	1.48 (1.33, 1.66)^**^
36–55	–	–	1.00
Sex			
Male	70.81	1	1.40 (1.29, 1.51)^**^
Female	–	–	1.00
Race			
The Hans	19.55	1	0.69 (0.59, 0.82)^**^
Other	–	–	1.00
Job			
Student, employee	0.60	1	1.07 (0.91, 1.25)
Self-employed	27.83	1	1.62 (1.35, 1.93)^**^
Retired and unemployed	–	–	1.00
Body mass index, kg/m^2^			
< 18.5	2.11	1	1.09 (0.97, 1.22)
18.5–23.9	0.51	1	0.97 (0.89, 1.06)
> 23.9	–	–	1.00
Chronic disease^b^			
Yes	51.58	1	1.93 (1.61, 2.31)^**^
No	–	–	1.00
Regular physical activity^c^			
Yes	61.54	1	1.37 (1.26, 1.48)^**^
No	–	–	1.00
Smoking status^d^			
Current smoker	1.41	1	1.08 (0.95, 1.23)
Former smoker	5.16	1	1.21 (1.03, 1.43)^*^
Non-smoker	–	–	1.00
Drinking status^e^			
Current drinker	0.01	1	1.00 (0.89, 1.11)
Former drinker	7.14	1	1.17 (1.04, 1.31)^*^
Non-drinker	–	–	1.00
Contact history^f^			
Yes	7.022	1	1.15 (1.04, 1.28)^*^
No	–	–	1.00
Suspicion of SARS-CoV-2 infection			
Yes	22.64	1	1.66 (1.35, 2.04)^**^
No	–	–	1.00
Front-line medical personnel			
Yes	111.09	1	2.12 (1.85, 2.44)^**^
No	–	–	1.00
Gatherings & meetings^g^			
Yes	18.26	1	0.83 (0.76, 0.90)^**^
No	–	–	1.00
Meeting relatives or friends coming from Hubei in the past month			
Yes	7.45	1	1.23 (1.06, 1.43)^*^
No	–	–	1.00
Exposure to wild animals			
Yes	5.61	1	1.31 (1.05, 1.63)^*^
No	–	–	1.00
Wearing masks			
Yes	40.16	1	0.71 (0.64, 0.79)^**^
No	–	–	1.00
Knowledge about personal protective measures			
Yes	1272.24	1	0.25 (0.23, 0.27)^**^
No	–	–	1.00
Present symptoms of SARS-CoV-2 infection^h^			
Yes	12.63	1	1.40 (1.16, 1.68)^**^
No	–	–	1.00

Abbreviations: CI, confidence interval

^a^All variables were used in the ordinal multivariate logistic regression. The participants with severe anxiety were selected as the reference frame

^b^Including hypertension, hyperlipidemia, diabetes, asthma, chronic obstructive pulmonary disease (COPD), chronic bronchitis, heart disease, gout, thyroid nodules, thyroid cancer, and lung cancer

^c^Regular physical activity was defined as regular exercise within the recent six months

^d^Current smoker was defined as an adult who has smoked 100 cigarettes in his or her lifetime and who currently smokes cigarettes; former smoker was defined as an adult who has smoked at least 100 cigarettes in his or her lifetime but who had quit smoking at the time of interview; while non-smoker was defined as an adult who has never smoked, or who has smoked less than 100 cigarettes in his or her lifetime

^e^Current drinker was defined as at least 12 drinks in the past year; former drinker was defined as at least 12 drinks in any one year in lifetime but no drinks in past year; while non-drinker was defined as fewer than 12 drinks in lifetime

^f^Close contact with a confirmed or suspected case of COVID-19 without taking precautions

^g^Been to a company meeting or a family dinner in the last two weeks

^h^Including fever, cough, runny nose, sore throat, shortness of breath, fatigue, nasal congestion, headache, vomiting and diarrhea

^*^p < .05

^**^p < .001

**Fig. 2:**
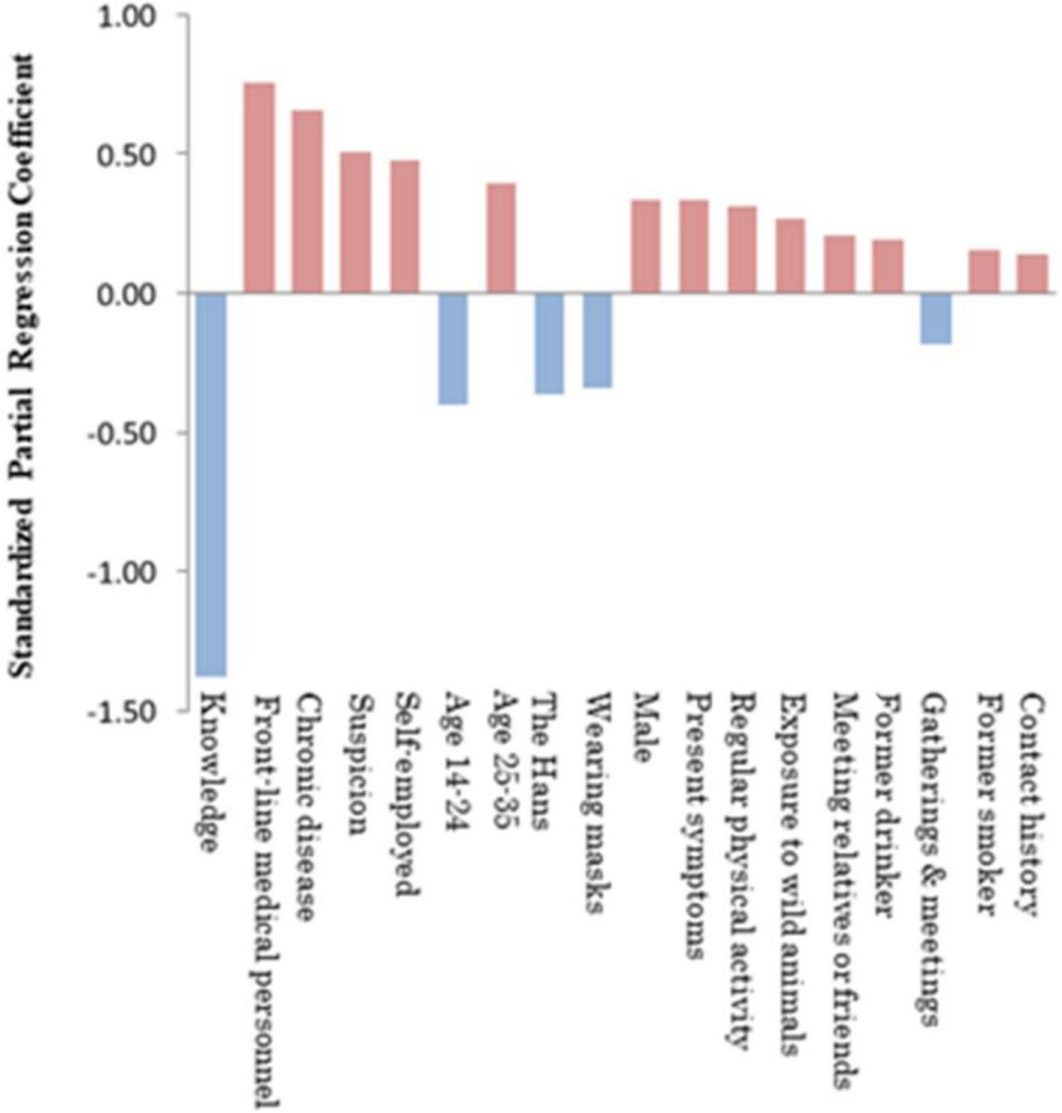
Standardized Partial Regression Coefficient of Factors. Abbreviations: Meeting relatives or friends: Meeting relatives or friends coming from Hubei in the past month; Present symptoms: Present symptoms of SARS-CoV-2 infection; Suspicion: Suspicion of SARS-CoV-2 infection; Knowledge: Knowledge about personal protective measures. Chronic diseases included hypertension, hyperlipidemia, diabetes, asthma, chronic obstructive pulmonary disease (COPD), chronic bronchitis, heart disease, gout, thyroid nodules, thyroid cancer, and lung cancer. Regular physical activity was defined as regular exercise within the recent six months. Current smoker was defined as an adult who has smoked 100 cigarettes in his or her lifetime and who currently smokes cigarettes; former smoker was defined as an adult who has smoked at least 100 cigarettes in his or her lifetime but who had quit smoking at the time of interview; while non-smoker was defined as an adult who has never smoked, or who has smoked less than 100 cigarettes in his or her lifetime. Current drinker was defined as at least 12 drinks in the past year; former drinker was defined as at least 12 drinks in any one year in lifetime but no drinks in past year; while non-drinker was defined as fewer than 12 drinks in lifetime. Contact history was defined as close contact with a confirmed or suspected case of COVID-19 without taking precautions. Gatherings and meetings were defined as been to a company meeting or a family dinner in the last two weeks. Present symptoms of SARS-CoV-2 infection included fever, cough, runny nose, sore throat, shortness of breath, fatigue, nasal congestion, headache, vomiting and diarrhea

We evaluated the contribution of each factor to the model by calculating the standardized coefficients, which can make the effect of factors on anxiety comparable (Fig. 2). Knowledge about personal protective measures (β = − 1.38) contributed most to the risk of anxiety, following by front-line medical personnel (β = 0.75), chronic disease (β = 0.66), suspicion of SARS-CoV-2 infection (β = 0.51), self-employed (β = 0.48), age (14–24: β = − 0.40; 25–35: β = 0.39), race (β = − 0.37), wearing masks (β = − 0.37), sex (β = 0.34), present symptoms of SARS-CoV-2 infection (β = 0.33), regular physical activity (β = 0.31), exposure to wild animals (β = 0.27), meeting relatives or friends coming from Hubei in the past month (β = 0.21), former smoking (β = 0.19), gatherings and meetings (β = − 0.19), drinking (β = 0.15), and contact history (β = 0.14).

### Risk score development

The risk score was created using the single score from the statistically significant 17 factors in the final model (Table S3). All participants were further divided into four groups by the risk score of 0, 1–2, 3, and ≥ 4. Anxiety index score and the percentage of anxiety significantly increased with the risk score group of 0 (very low risk), 1–2 (low risk), 3 (moderate risk), and ≥ 4 (high risk) (average anxiety index score: 35.8, 35.4, 37.3 and 45.0 respectively; percentage of anxiety: 7.6, 9.3, 17.9 and 38.9%, respectively) (Figure S1 and Figure S2).

Continuous analysis by linear regression models showed that each one-point increase in risk score was associated with a 2.97 (95% CI: 2.86, 3.09) increase in anxiety index score. In categorical analysis, we also found the moderate risk group and high risk group had a 1.44 (95% CI: 0.27, 2.61) and 9.18 (95% CI: 8.04, 10.33) increase in anxiety index score, when compared with the very low risk group.

**Fig. 3:**
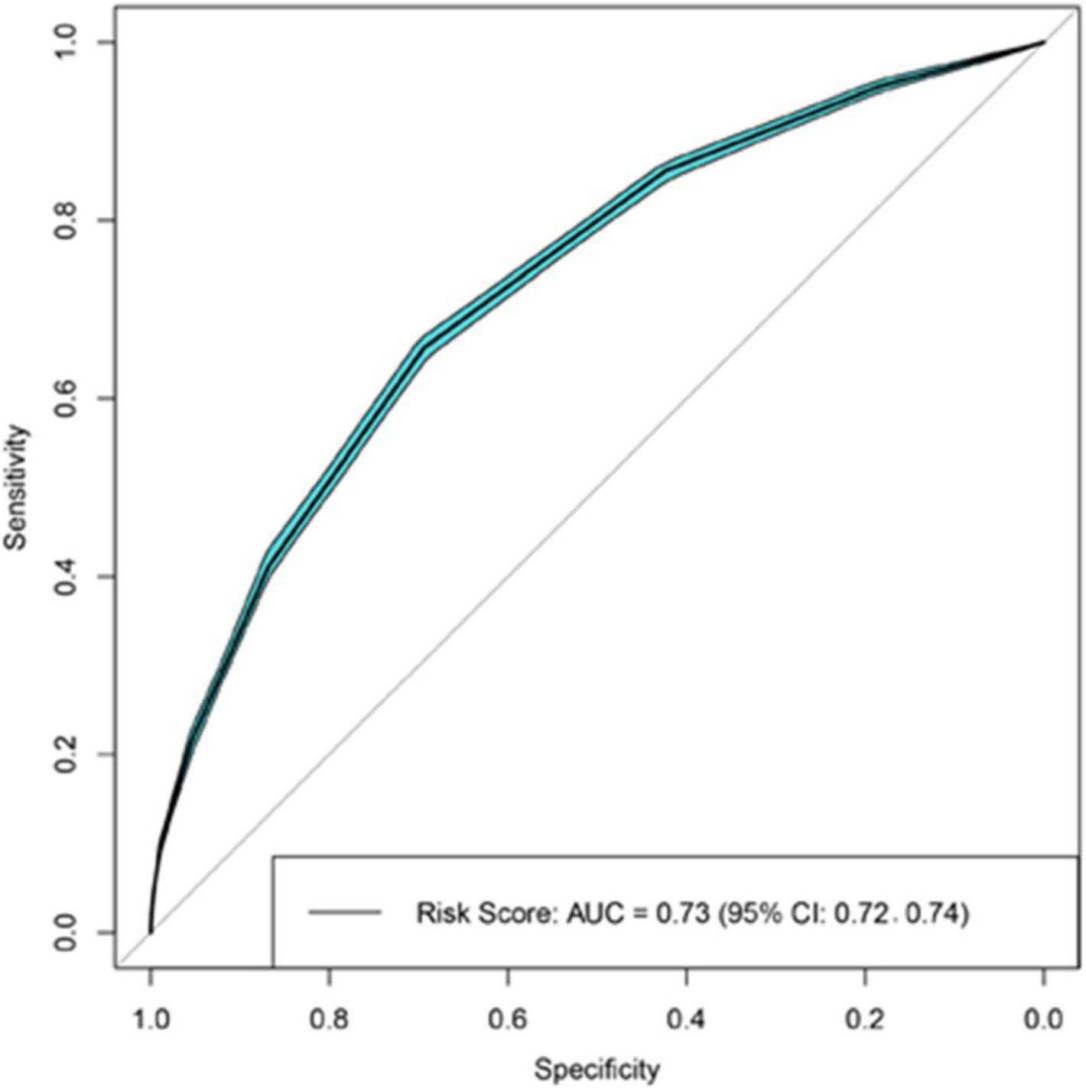
Description of the Developed Risk Score Prediction Performance for Anxiety by AUC. Abbreviations: AUC, area under the Receive Operating Characteristic Curve. Solid black line represents results for model including risk score, with light green area indicating the 95% CIs

Compared with those in very low risk, participants in low risk, moderate risk, and high risk group had 26% (95% CI: − 7.4, 72%), 172% (95% CI: 100, 270%), and 733% (95% CI: 516, 1026%) higher risk of higher anxiety level respectively (Table 4). We further attempt to develop risk score as a potential predictor of anxiety by generating ROC (Fig. 3). We found that the AUC was 0.73 (95% CI: 0.72, 0.74) for model with the developed risk score, with the cut-off point of 3.5.

**Table 4:** Analyses of the Association Between Anxiety Level and Risk Score Group (*n* = 19,802)

Risk score	Number of participants (%)					Odds ratio (95% CI)^a^
	All participants (*n* = 19,802)	Normal (*n* = 15,277)	Mild anxiety (*n* = 2157)	Moderate anxiety (*n* = 1268)	Severe anxiety (*n* = 1100)	
0	632 (3.2)	584 (2.9)	46 (0.2)	2 (0.0)	0 (0.0)	1.00
1–2	6493 (32.8)	5889 (29.7)	451 (2.3)	109 (0.6)	44 (0.2)	1.26 (0.93, 1.72)
3	5030 (25.4)	4130 (20.9)	507 (2.6)	218 (1.1)	175 (0.9)	2.72 (2.00, 3.70)^**^
≥ 4	7647 (38.6)	4674 (23.6)	1153 (5.8)	939 (4.7)	881 (4.4)	8.33 (6.16, 11.26)^**^

Abbreviations: CI, confidence interval

^a^Ordinal multivariate logistic regression was used and the participants with severe anxiety was selected as the reference frame

**p < .001

## Discussion

In this study, we found that those who were front-line medical personnel, suffered from chronic disease, with present symptoms of SARS-CoV-2 infection or contact history had 112, 93, 40 and 15% increased risk of higher anxiety level; while those with knowledge about personal protective measures or wore masks had 75 and 29% lower risk of higher anxiety level respectively. We developed a risk score to assess the total effect of observed significant factors on anxiety and found that each one increase of the risk score was associated with increase in anxiety index score, as well as increased risk of anxiety.

There are over 127 million confirmed cases of COVID-19 across the globe. In addition to physical injuries caused by SARS-CoV-2 infections, psychological injuries should also be concerned. As COVID-19 is a novel coronavirus disease, there was few evidence on the risk and protective factors for anxiety. Several factors associated with anxiety symptoms were reported in several studies [20–22]. For example, physical exercise and smoking status were linked to the risk of anxiety [20, 21]. Moreover, considering the particularities of the COVID-19 pandemic, we included several factors associated with COVID-19 infections (e.g. exposure to wild animals, gatherings and meetings) when exploring the risk and protective factors for anxiety symptoms amid COVID-19. Our finding observed the anxiety in a Chinese population during the COVID-19 pandemic and helped to reveal anxiety-related factors including front-line medical personnel, individuals with contact history and so on. It emphasizes the importance of psychosocial intervention to reduce the anxiety during the COVID-19, especially among individuals with chronic diseases and front-line medical personnel.

Compared with previous studies, similar information may be derived by previous experiences with coronavirus infections. Front-line medical personnel may develop psychiatric disorders after coping with stressful community events [13, 23–26]. This could be attributed to medical workers facing enormous pressure, including a high risk of infection and inadequate protection from contamination, being overworked, experiencing frustration, discrimination, isolation, patients with negative emotions, a lack of contact with their families, and exhaustion [27]. Some demographic factors may also influence mental health during the COVID-19 pandemic. Individuals with contact history had an increased risk of anxiety for the reason that they not only had to undergo the high possibility of being infectious, but also had to experience alienation in their neighborhood resulting in a hardened mental impact. Particular precautionary measures (e.g., wearing masks) were associated with a lower psychological impact of the outbreak and lower levels of stress, and anxiety [28], since the adoption of self-protective measures can effectively reduce the risk of infection.

We developed a risk score to assess the total effect of factors on anxiety. The results from linear regression models and logistic models consistently showed the significant association between the developed risk score and anxiety index score/disorder. The AUC of 0.73 confirmed the risk score on prediction of anxiety. In addition, the cut-off point of 3.5 indicated that individual who was with more than three observed significant related factors had higher risk of suffering from anxiety during the COVID-19 pandemic. The risk factors (e.g., front-line medical personnel, exposure to wild animals, contact history, and chronic disease) are related with elevated risk scores (Figure S3). Particular precautionary measures (e.g., wearing masks) and knowledge about personal protective measures may have a protective effect on risk scores (Figure S3).

There are several tools to assess anxiety including Hamilton anxiety scale, Generalized Anxiety Disorder (GAD-7) and The Coronavirus Anxiety Scale (CAS). Compared with previous self-report anxiety-like measures (i.e. GAD-7 and CAS), the Chinese version of GAD-7 screening tool was used to assess for anxiety symptoms [29], with increasing scores indicating more severe functional impairments as a result of anxiety [30]. The GAD-7 focuses more on dysfunction and disability than SAS. However, both of which are used to assess for anxiety symptoms and represent a reasonable cut point for identifying cases of different levels of anxiety. The CAS, which is a brief mental health screener to identify probable cases of dysfunctional anxiety associated with the COVID-19 crisis [31]. The CAS discriminates well between persons with and without dysfunctional anxiety using an optimized cut score of ≥9. The CAS’ items center on anxiety and trauma related reactions and distressing bodily symptoms, make them highly relevant to somatic symptom and related disorders. Moreover, the CAS was shown to measure anxiety symptoms in similar ways across different populations, which cannot be verified on SAS.

We observed several notable risk factors associated with elevated anxiety in the Chinese population during the COVID-19 outbreak. For example, those with chronic disease were observed to have higher risk for anxiety, which were similar with those reported in the previous studies [3, 12]. The World Health Organization (WHO) has reported that patients with pre-existing noncommunicable diseases, including cardiovascular disease, chronic respiratory disease, diabetes, and cancer, are at increased risk of severe illness from COVID-19 [32]. This might play an important role in the development of anxiety. Moreover, the general public with game exposure had a greater likelihood of anxiety during the pandemic. Exposure to live commercial and private poultry is a potential risk factor for infection with novel influenza viruses [33].

Most of the studies used the Zung scale were Chinese studies, while some studies have reported that Pakistani [34] and Malaysia [35] used Zung’s scale to assess anxiety of the COVID-19 outbreak among university students. They found that being female and younger age are risk factors. The risk score we developed can help to easily screen out individuals with high risk of anxiety through simple questions, in order to take reasonable psychological interventions in time. Zung’s scale is approved for large sample sizes for Chinese populations, though to further the validity of the scale it could also be expanded to large sample sizes in other countries.

This study has several strengths. First, the sample size of our cross-sectional study was considerably large, which enabled us to estimate the association between uncommon risks and anxiety with sufficient statistical power. Second, we performed multiple methods to identify and confirm the anxiety-related risks, and developed a simple way to assess anxiety during the special period. There are also some limitations. Similar with most previous psychological studies, the data we collected is based on self-report online questionnaires, which can cause response bias although it was easy to obtain. However, we have carried out quality control including setting up similar questions in the questionnaire and performing logical checks to ensure the reliability of the data. Considering that the questionnaire was distributed online, the study cannot reach the participants without smartphone and unequal distribution of participants across provinces. Although we observed the significant associations between some risks and anxiety, we should also note that the data cannot be used to infer causality due to the cross-sectional design. Considering the differences of mobility and distribution of anxiety, as well as the prevention and control measures for protecting from COVID-19 in different countries, whether the results can be taken and applied to other regions or populations is in need of more evidence.

## Conclusions

The findings revealed protective and risk factors associated with anxiety and developed a practical and simple score to identify individuals who are at risk of anxiety. This research offered preliminary support for relieving anxiety as an acceptable selective preventive intervention for people during COVID-19 pandemic. The generalizability of our study is limited and further studies are needed to investigate the protective and risk factors for anxiety during COVID-19 pandemic.

## Supplementary Information

**Additional file 1 Table S1.** Characteristics of Participants with Different Levels of Anxiety (*n* = 19,802). **Table S2.** The First Ordinal Multivariable Logistic Regression Analyses of the Association Between Characteristic and Anxiety Level (*n* = 19,802). **Table S3.** Scoring of the Variables in the Anxiety Scoring System. **Figure S1.** Anxiety Index Score in Four Risk Score Groups. **Figure S2.** Anxiety Level of Participants in Different Risk Score Groups. **Figure S3.** Percentages of Each Risk Factor Among High Risk Group (Participants with the Risk Score ≥ 4).

## Data Availability

The datasets generated and/or analyzed during the current study are not publicly available due legal restriction but are available from the corresponding author on reasonable request.

## Abbreviations

COVID-19: Corona Virus Disease 2019
OR: Odds radio
CI: Confidence interval
SAS: Self-Rating Anxiety Scale
SD: Standard deviation
AUC: Area under curve
