# Supplementary material for: Risk and protective factors for anxiety during COVID-19 pandemic
Source: BMC Public Health. 2021 Jun 4;21:1063. doi: 10.1186/s12889-021-11118-8 (PMC8177261; doi:10.1186/s12889-021-11118-8)
Supplement: Supplementary file 1 — Additional file 1 Table S1. Characteristics of Participants with Different Levels of Anxiety (n = 19,802). Table S2. The First Ordinal Multivariable Logistic Regression Analyses of the Association Between Characteristic and Anxiety Level (n = 19,802). Table S3. Scoring of the Variables in the Anxiety Scoring System. Figure S1. Anxiety Index Score in Four Risk Score Groups. Figure S2. Anxiety Level of Participants in Different Risk Score Groups. Figure S3. Percentages of Each Risk Factor Among High Risk Group (Participants with the Risk Score ≥ 4). [file 12889_2021_11118_MOESM1_ESM.docx]

**SUPPLEMENTARY APPENDIX**

*Supplement to:*

*Zhong J.Y., Zhong C.H., Qiu L., Li J.Y., Lai J.Y., Lu W.F., Wang S.G., Zhong J.C., Zhao J., Zhou Y.*

*Title: Risk and Protective Factors for Anxiety During COVID-19 Pandemic*

**Table of contents**

| **Contents** | **Page** |
| --- | --- |
| Table S1 Characteristics of Participants with Different Levels of Anxiety (*n* = 19,802) | 2 |
| Table S2 The First Ordinal Multivariable Logistic Regression Analyses of the Association Between Characteristic and Anxiety Level (*n* = 19,802) | 5 |
| Table S3 Scoring of the Variables in the Anxiety Scoring System | 8 |
| Figure S1 Anxiety Index Score in Four Risk Score Groups | 11 |
| Figure S2 Anxiety Level of Participants in Different Risk Score Groups | 12 |
| Figure S3 Percentages of Each Risk Factor Among High Risk Group (Participants with the Risk Score ≥ 4) | 13 |

**Table S1 Characteristics of Participants with Different Levels of Anxiety (*n* = 19,802)**

| Characteristics, *n* (%) | Anxiety level | | | |
| --- | --- | --- | --- | --- |
|  | Normal  (*n* = 15,277) | Mild  anxiety  (*n* = 2,157) | Moderate anxiety  (*n* = 1,268) | Severe anxiety  (*n* = 1,100) |
| Age, year |  |  |  |  |
| 14-24 | 9,712 (63.6) | 1,115 (51.7) | 496 (39.1) | 307 (27.9) |
| 25-35 | 3,751 (24.6) | 798 (37.0) | 603 (47.6) | 594 (54.0) |
| 36-55 | 1,814 (11.9) | 244 (11.3) | 169 (13.3) | 199 (18.1) |
| Male | 7,270 (47.6) | 1,272 (59.0) | 862 (68.0) | 717 (65.2) |
| Body mass index |  |  |  |  |
| < 18.5 | 3,306 (21.6) | 468 (21.7) | 226 (17.8) | 232 (21.1) |
| 18.5-23.9 | 8,956 (58.6) | 1,249 (57.9) | 745 (58.8) | 603 (54.8) |
| > 23.9 | 3,015 (19.7) | 440 (20.4) | 297 (23.4) | 265 (24.1) |
| Race |  |  |  |  |
| The Hans | 14,841 (97.1) | 2,037 (94.4) | 1,170 (92.3) | 1,027 (93.4) |
| Other | 436 (2.9) | 120 (5.6) | 98 (7.7) | 73 (6.6) |
| Smoking status^a^ |  |  |  |  |
| Current smoker | 1,154 (7.6) | 231 (10.7) | 169 (13.3) | 150 (13.6) |
| Former smoker | 468 (3.1) | 111 (5.1) | 94 (7.4) | 91 (8.3) |
| Non-smoker | 13,655(89.4) | 1,815 (84.1) | 1,005 (79.3) | 859 (78.1) |
| Drinker status^b^ |  |  |  |  |
| Current drinker | 2,289 (15.0) | 358 (16.6) | 230 (18.1) | 180 (16.4) |
| Former drinker | 1,437 (9.4) | 294 (13.6) | 204 (16.1) | 181 (16.5) |
| Non-drinker | 11,551 (75.6) | 1,505 (69.8) | 834 (65.8) | 739 (67.2) |
| Job |  |  |  |  |
| Student or employee | 13,138 (86.0) | 1,727 (80.1) | 961 (75.8) | 822 (74.7) |
| Self-employed | 1,311 (8.6) | 324 (15.0) | 236 (18.6) | 225 (20.5) |
| Retired and unemployed | 828 (5.4) | 106 (4.9) | 71 (5.6) | 53 (4.8) |
| Front-line medical personnel | 467 (3.1) | 144 (6.7) | 155 (12.2) | 198 (18.0) |
| In Hubei Province in the past month | 616 (4.0) | 155 (7.2) | 112 (8.8) | 133 (12.1) |
| Meeting relatives or friends coming from Hubei in the past month | 719 (4.7) | 160 (7.4) | 133 (10.5) | 147 (13.4) |
| Quarantine^c^ | 897 (5.9) | 122 (5.7) | 77 (6.1) | 79 (7.2) |
| Exposure to wild animals | 195 (1.3) | 72 (3.3) | 77 (6.1) | 92 (8.4) |
| Gatherings & meetings^d^ | 4,785 (31.3) | 654 (30.3) | 325 (25.6) | 263 (23.9) |
| Wearing masks | 13,803 (90.4) | 1,849 (85.7) | 1,046 (82.5) | 923 (83.9) |
| Regular physical activity^e^ | 7,991 (52.3) | 1,223 (56.7) | 885 (69.8) | 853 (77.5) |
| Suspicionof SARS-CoV-2 infection | 340 (2.2) | 95 (4.4) | 75 (5.9) | 61 (5.5) |
| Contact history^f^ | 2,357 (15.4) | 340 (15.8) | 196 (15.5) | 111 (10.1) |
| Knowledge about personal protective measures | 13,052 (85.4) | 1,351 (62.6) | 584 (46.1) | 431 (39.2) |
| Present symptoms of SARS-CoV-2 infection^g^ | 548 (3.6) | 119 (5.5) | 87 (6.9) | 73 (6.6) |
| Chronic disease^h^ | 263 (1.7) | 86 (4.0) | 95 (7.5) | 114 (10.4) |

^a^Current smoker was defined as an adult who has smoked 100 cigarettes in his or her lifetime and who currently smokes cigarettes; former smoker was defined as an adult who has smoked at least 100 cigarettes in his or her lifetime but who had quit smoking at the time of interview; while non-smoker was defined as an adult who has never smoked, or who has smoked less than 100 cigarettes in his or her lifetime.

^b^Current drinker was defined as at least 12 drinks in the past year; former drinker was defined as at least 12 drinks in any one year in lifetime but no drinks in past year; while non-drinker was defined as fewer than 12 drinks in lifetime.

^c^Been or are in quarantine for this outbreak, including mandatory isolation and self isolation at home/hotel)

^d^Been to a company meeting or a family dinner in the last two weeks.

^e^Regular physical activity was defined as regular exercise within the recent six months.

^f^Close contact with a confirmed or suspected case of COVID-19 without taking precautions.

^g^Including fever, cough, runny nose, sore throat, shortness of breath, fatigue, nasal congestion, headache, vomiting and diarrhea.

^h^Including hypertension, hyperlipidemia, diabetes, asthma, chronic obstructive pulmonary disease (COPD), chronic bronchitis, heart disease, gout, thyroid nodules, thyroid cancer, and lung cancer.

**Table S2 The First Ordinal Multivariable Logistic Regression Analyses of the Association Between Characteristic and Anxiety Level (*n* = 19,802)**

| Characteristics | *t*-value | Odds ratio (95% CI)^a^ |
| --- | --- | --- |
| Age, year |  |  |
| 14-24 | 46.29 | 0.67 (0.60, 0.75)^**^ |
| 25-35 | 47.46 | 1.48 (1.33, 1.66)^**^ |
| 36-55 | - | 1.00 |
| Sex |  |  |
| Male | 70.39 | 1.40 (1.29, 1.51)^**^ |
| Female | - | 1.00 |
| Body mass index |  |  |
| < 18.5 | 2.12 | 1.09 (0.97, 1.22) |
| 18.5-23.9 | 0.50 | 0.97 (0.89, 1.06) |
| > 23.9 | - | 1.00 |
| Race |  |  |
| The Hans | 19.34 | 0.70 (0.59, 0.82)^**^ |
| Other | - | 1.00 |
| Smoking status^b^ |  |  |
| Current smoker | 1.57 | 1.09 (0.96, 1.24) |
| Former smoker | 5.31 | 1.22 (1.03, 1.44)^*^ |
| Non-smoker | - | 1.00 |
| Drinker status^c^ |  |  |
| Current drinker | 0.013 | 0.99 (0.89, 1.11) |
| Former drinker | 6.99 | 1.17 (1.04, 1.31)^*^ |
| Non-drinker | - | 1.00 |
| Job |  |  |
| Student or employee | 0.63 | 1.07 (0.91, 1.25) |
| Self-employed | 27.89 | 1.62 (1.35, 1.93)^**^ |
| Retired and unemployed | - | 1.00 |
| Front-line medical personnel | 111.06 | 2.15 (1.86, 2.47)^**^ |
| In Hubei Province in the past month | 0.47 | 0.94 (0.78, 1.13) |
| Meeting relatives or friends coming from Hubei in the past month | 8.11 | 1.29 (1.08, 1.54)^*^ |
| Quarantine^d^ | 2.43 | 0.88 (0.75, 1.03) |
| Exposure to wild animals | 6.09 | 1.32 (1.06, 1.65)^*^ |
| Gatherings & meetings^e^ | 17.95 | 0.83 (0.76, 0.91)^**^ |
| Wearing masks | 39.07 | 0.71 (0.64, 0.79)^**^ |
| Regular physical activity^f^ | 61.54 | 1.37 (1.26, 1.48)^**^ |
| Suspicion of SARS-CoV-2 infection | 23.88 | 1.68 (1.37, 2.07)^**^ |
| Contact history^g^ | 7.41 | 1.16 (1.04, 1.28)^*^ |
| Knowledge about personal protective measures | 1,268.47 | 0.25 (0.23, 0.27)^**^ |
| Present symptoms of SARS-CoV-2 infection^h^ | 13.45 | 1.41 (1.17, 1.69)^**^ |
| Chronic disease^i^ | 52.52 | 1.94 (1.62, 2.32)^**^ |

^a^All variables were used in the ordinal multivariate logistic regression. The participants with severe anxiety was selected as the reference frame.

^b^Current smoker was defined as an adult who has smoked 100 cigarettes in his or her lifetime and who currently smokes cigarettes; former smoker was defined as an adult who has smoked at least 100 cigarettes in his or her lifetime but who had quit smoking at the time of interview; while non-smoker was defined as an adult who has never smoked, or who has smoked less than 100 cigarettes in his or her lifetime.

^c^Current drinker was defined as at least 12 drinks in the past year; former drinker was defined as at least 12 drinks in any one year in lifetime but no drinks in past year; while non-drinker was defined as fewer than 12 drinks in lifetime.

^d^Been or are in quarantine for this outbreak, including mandatory isolation and self isolation at home/hotel)

^e^Been to a company meeting or a family dinner in the last two weeks.

^f^Regular physical activity was defined as regular exercise within the recent six months.

^g^Close contact with a confirmed or suspected case of COVID-19 without taking precautions.

^h^Including fever, cough, runny nose, sore throat, shortness of breath, fatigue, nasal congestion, headache, vomiting and diarrhea.

^i^Including hypertension, hyperlipidemia, diabetes, asthma, chronic obstructive pulmonary disease (COPD), chronic bronchitis, heart disease, gout, thyroid nodules, thyroid cancer, and lung cancer.

^*^*p* < .05

^**^*p* < .001

**Table S3 Scoring of the Variables in the Anxiety Scoring System**

| Variable | Score |
| --- | --- |
| Age, year |  |
| 14-24 | 0 |
| 25-55 | 1 |
| Sex |  |
| Male | 1 |
| Female | 0 |
| Race |  |
| The Hans | 0 |
| Other | 1 |
| Job |  |
| Student, employee | 0 |
| Self-employed | 1 |
| Retired and unemployed | 0 |
| Chronic disease^a^ |  |
| Yes | 1 |
| No | 0 |
| Regular physical activity^b^ |  |
| Yes | 1 |
| No | 0 |
| Smoking status^c^ |  |
| Current smoker | 0 |
| Former smoker | 1 |
| Non-smoker | 0 |
| Drinking status^d^ |  |
| Current drinker | 0 |
| Former drinker | 1 |
| Non-drinker | 0 |
| Contact history^e^ |  |
| Yes | 1 |
| No | 0 |
| Suspicion of SARS-CoV-2 infection |  |
| Yes | 1 |
| No | 0 |
| Front-line medical personnel |  |
| Yes | 1 |
| No | 0 |
| Gatherings & meetings^f^ |  |
| Yes | 0 |
| No | 1 |
| Meeting relatives or friends coming from Hubei in the past month |  |
| Yes | 1 |
| No | 0 |
| Exposure to wild animals |  |
| Yes | 1 |
| No | 0 |
| Present symptoms of SARS-CoV-2 infection^g^ |  |
| Yes | 1 |
| No | 0 |
| Knowledge about personal protective measures |  |
| Yes | 0 |
| No | 1 |
| Wearing masks |  |
| Yes | 0 |
| No | 1 |

^a^Including hypertension, hyperlipidemia, diabetes, asthma, chronic obstructive pulmonary disease (COPD), chronic bronchitis, heart disease, gout, thyroid nodules, thyroid cancer, and lung cancer.

^b^Regular physical activity was defined as regular exercise within the recent six months.

^c^Current smoker was defined as an adult who has smoked 100 cigarettes in his or her lifetime and who currently smokes cigarettes; former smoker was defined as an adult who has smoked at least 100 cigarettes in his or her lifetime but who had quit smoking at the time of interview; while non-smoker was defined as an adult who has never smoked, or who has smoked less than 100 cigarettes in his or her lifetime.

^d^Current drinker was defined as at least 12 drinks in the past year; former drinker was defined as at least 12 drinks in any one year in lifetime but no drinks in past year; while non-drinker was defined as fewer than 12 drinks in lifetime.

^e^Close contact with a confirmed or suspected case of COVID-19 without taking precautions.

^f^Been to a company meeting or a family dinner in the last two weeks

^g^Including fever, cough, runny nose, sore throat, shortness of breath, fatigue, nasal congestion, headache, vomiting and diarrhea.


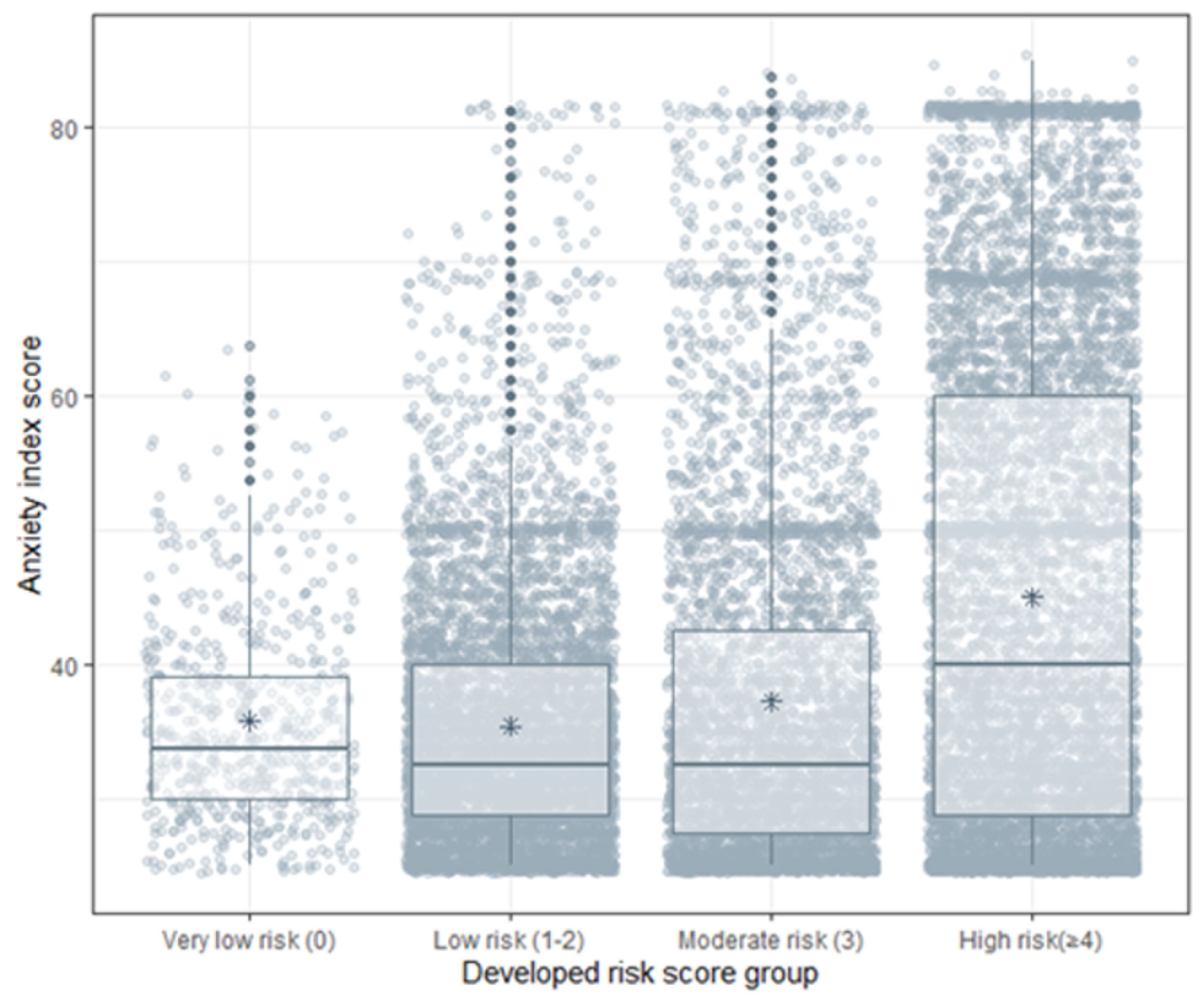


**Figure S1Anxiety Index Score in Four Risk Score Groups.** Asterisks indicate the mean; horizontal line indicates the median; greypoints represent subjects;black points identify the more extreme observations; lower and upper edges of the boxes represent the 25th and 75th percentiles (interquartile range [IQR]). The upper and lower extremes of the whiskers are defined as 1.5 × IQR above and 1.5 × IQR below the 75th and 25th percentiles.


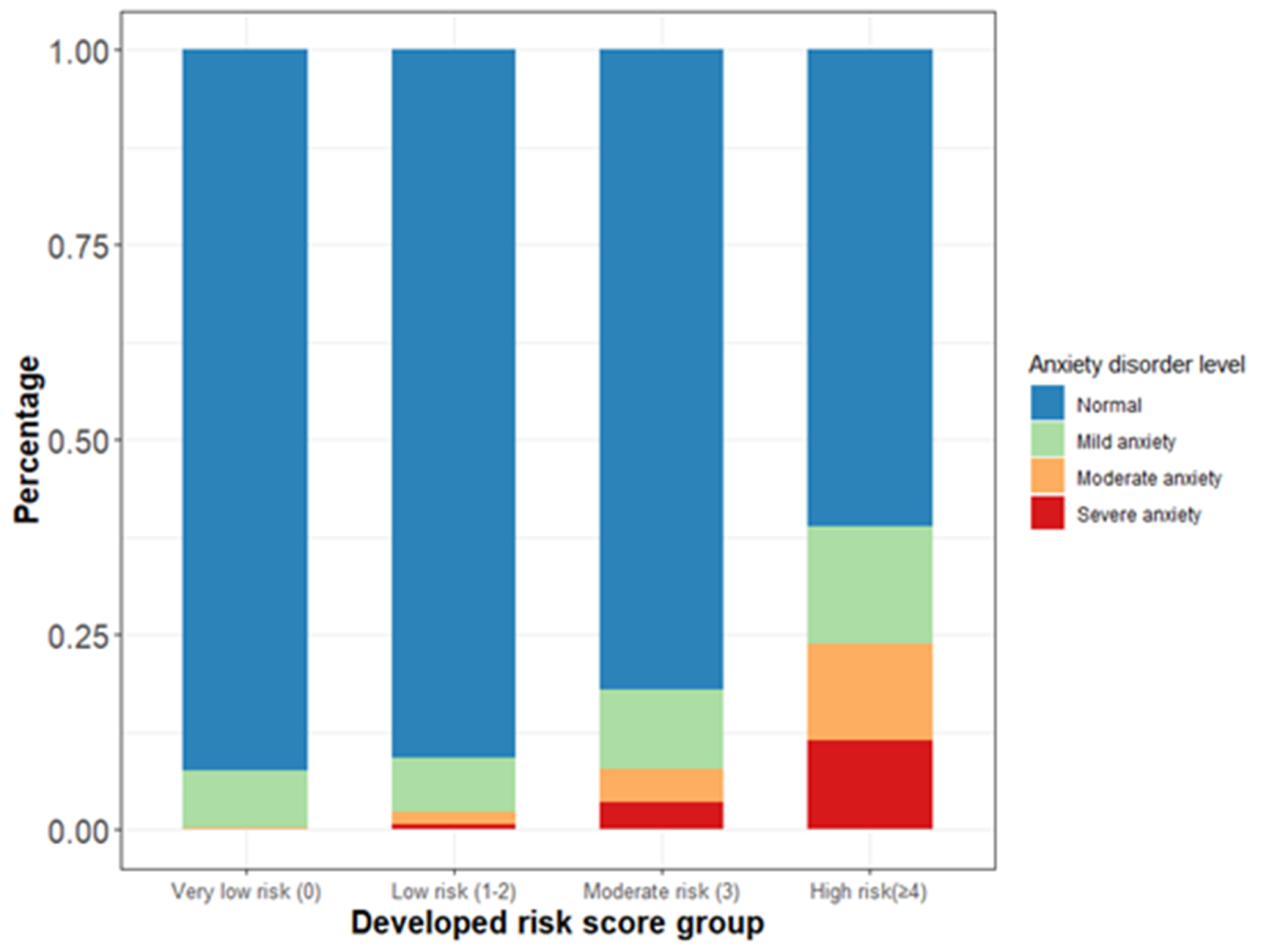


**Figure S2 Anxiety Level of Participants in Different Risk Score Groups.**

**
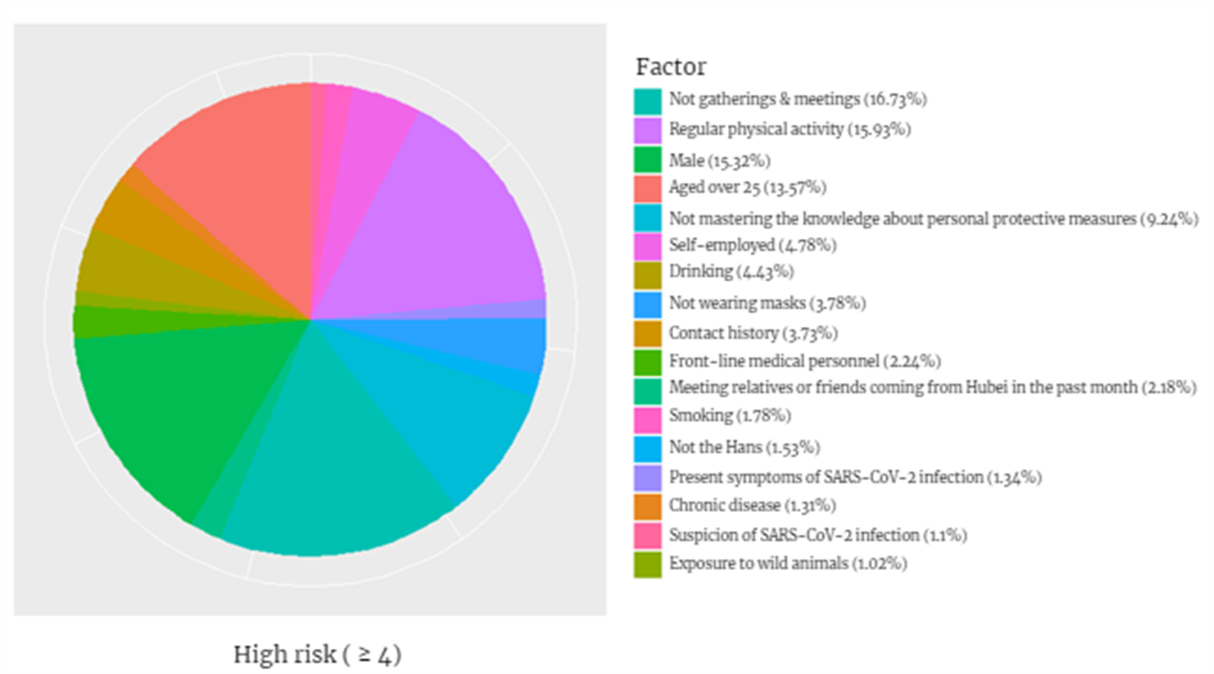
**

**Figure S3 Percentages of Each Risk Factor Among High Risk Group (Participants with the Risk Score ≥ 4).**
